# Etiology of language network changes during recovery of aphasia after stroke

**DOI:** 10.1038/s41598-018-19302-4

**Published:** 2018-01-16

**Authors:** Casper A. M. M. van Oers, H. Bart van der Worp, L. Jaap Kappelle, Mathijs A. H. Raemaekers, Willem M. Otte, Rick M. Dijkhuizen

**Affiliations:** 1 Department of Neurology and Neurosurgery, Brain Center Rudolf Magnus, University Medical Center Utrecht and Utrecht University, 3584 CX Utrecht, The Netherlands; 2 Biomedical MR Imaging and Spectroscopy Group, Center for Image Sciences, University Medical Center Utrecht and Utrecht University, 3584 CX Utrecht, The Netherlands; 3 Department of Neurology, Amphia Hospital, 4818 CK Breda, The Netherlands; 4 Department of Pediatric Neurology, Brain Center Rudolf Magnus, University Medical Center Utrecht and Utrecht University, 3584 CX Utrecht, The Netherlands

## Abstract

Knowledge of spatiotemporal patterns of language network changes may help in predicting outcome in aphasic stroke patients. Here we assessed language function and performed functional MRI four times during one year to measure language network activation and cerebrovascular reactivity (with breath-holding) in twelve left-hemispheric stroke patients, of whom two dropped out before the final measurement, and eight age-matched controls. Language outcome was related to increase of activation in left and right posterior inferior temporal gyrus over the first year, while activation increase in right inferior frontal gyrus was inversely correlated to language recovery. Outcome prediction improved by addition of early language-induced activation of the left posterior inferior temporal gyrus to a regression model with baseline language performance as first predictor. Variations in language-induced activation in right inferior frontal gyrus were primarily related to differences in vascular reactivity. Furthermore, several language-activation changes could not be linked to alterations in language proficiency nor vascular reactivity, and were assumed to be caused by unspecified intersession variability. In conclusion, early functional neuroimaging improves outcome prediction of aphasia after stroke. Controlling for cerebrovascular reactivity and unspecified intersession variability may result in more accurate assessment of the relationship between activation pattern shifts and function after stroke.

## Introduction

Each year, around 15 million people are affected by stroke worldwide^1^. After dementia, it is the second leading cause of disability. Roughly a third of stroke patients are affected by aphasia^2^. Spontaneous recovery from aphasia after stroke is variable, and prediction of individual outcome is difficult^3^. Classical prediction models with conventional predictor variables, such as acute language deficit, lesion size and location, and patient characteristics, may however improve by incorporating brain activation during language performance in the subacute phase –when acute non-system specific changes have mostly dissolved– as a predictor variable^4^. The activation pattern within the brain’s language network has been shown to alter over time after stroke: left frontal parts of the language network often harbor increase of activation from the subacute to chronic phase, whereas activity in the right inferior frontal gyrus (IFG) decreases^5^. While the right IFG may have a negative influence on local processing in the chronic phase^6^, a positive contribution has been suggested during earlier phases of recovery^5,7^. Knowledge of the potential contribution of different left and right hemispheric language nodes at different stages of recovery is crucial for the understanding of changes of processing within the language network, which could further assist prediction of outcome in individual patients.

Functional MRI (fMRI) provides a practical tool for non-invasive whole-brain assessment of activation patterns during execution of language tasks. In fMRI, neuronal activity is inferred from a hemodynamic response. Since stroke patients with cerebrovascular dysfunction may have a significantly altered neuronal-hemodynamic coupling^8^, cerebrovascular reactivity is a critical factor in the sensitivity and specificity of fMRI-based detection of post-stroke network activity. Moreover, changes in vascular reactivity over time may be a serious confounder in the interpretation of serial fMRI studies. Although we have previously shown with a breath hold paradigm that no significant misinterpretation is expected in the chronic phase^9^, this remains unclear for earlier phases after stroke.

An additional confounder in (serial) fMRI studies is nonspecific intersession variability. Variations in fMRI-detected activation patterns have been observed between different language task sessions in healthy subjects and in aphasic patients, which were not related to actual changes in network properties^10^. Therefore, changes in activation measured from repetition of a single fMRI task may not necessarily reflect reorganization of the involved neural network.

In the current study, we aimed to predict language outcome at one year in a group of aphasic stroke patients by applying fMRI in the subacute phase and assessing the contribution of different parts of the brain to language performance in the subacute to chronic phase after stroke. A longitudinal fMRI study was conducted in patients with aphasia after stroke and healthy controls. Two language tasks, as well as a breath hold paradigm, were included at two weeks, three, six and twelve months after stroke. We hypothesized that (i) the extent of activity in left hemispheric language areas around two weeks after stroke as a predictor variable improves prediction of outcome at one year; (ii) the amount of language-induced activation in the left hemisphere is positively related to language performance, while activation of language areas in the right hemisphere is inversely related to language skill; and (iii) cerebrovascular reactivity and intersession variation of undetermined origin can confound the interpretation of changes of the fMRI signal over time.

## Methods

### Subjects

Patients were recruited at the stroke units of the University Medical Center Utrecht and the Sint Antonius Hospital Nieuwegein, The Netherlands. Inclusion criteria were: i) first-ever ischemic stroke in the territory of the left middle cerebral artery; ii) aphasia measurable on the Dutch version of the Aachen Aphasia Test (AAT)^11^; iii) mild to moderate disability (modified Rankin scale score ≤ 3)^12^; iv) right-handedness; and v) Dutch as native language. Exclusion criteria were; i) history of another neurological or psychiatric disease; ii) MRI-incompatible prosthesis; and iii) inability to perform the MRI paradigms due to the severity of aphasia or comorbidity.

The control subjects were matched for age, and level of education, by including the patients’ spouses, siblings, similarly aged other relatives or acquaintances. Exclusion criteria for controls were: i) neurological or psychiatric disease; ii) Dutch not being the native language; iii) MRI-incompatible prostheses; and iv) left handedness.

All methods were carried out in accordance with relevant guidelines and regulations. All experimental protocols were approved by the local Medical Ethical Review Board of the University Medical Center Utrecht. Written informed consent was obtained from all subjects.

### Data acquisition

#### Language testing

Patients were examined at four time points after stroke: within 2 weeks and at 3, 6, and 12 months. Control subjects were examined at the same time intervals. Language testing of the stroke patients consisted of the Dutch version of the AAT and the Boston Naming Task (BNT)^13^. Type (Broca, Wernicke, Anomic, Transcortical, Global) and severity of aphasia were defined with software included in the test battery. For further analysis, the sub-scores on the AAT and score on the BNT were transformed into Z-scores based on the scores of a reference population of Dutch aphasia patients^11^. Scores on the Token test, as part of the AAT, were inverted to have higher scores reflect better performance. The average of all Z-scores was used as a measure of language performance as described previously^9^. Language performance during online fMRI tasks was defined as the proportion of correct responses (language accuracy). Our previous study showed that ceiling effects restrict the usefulness of the online language accuracy. Therefore, to optimally relate the activation response associated with each fMRI task to language function, we averaged the Z-scores of subtasks of the AAT and BNT that matched most with the language processes essential for performance of the particular fMRI task (see below).

#### MRI

A 3 T Philips Achieva MR scanner and an eight-element RF head coil were used for all scans. At the first session around 2 weeks post-stroke, a T2-weighted multi-slice FLAIR scan (repetition time (TR)/echo time (TE)/inversion time (TI) = 11000/125/2800 ms, acquired voxel size = 0.65 × 0.94 × 4.0 mm^3^, reconstructed voxel size = 0.45 × 0.45 × 4.0 mm^3^, field-of-view (FOV) = 230 × 183 × 129 mm^3^, transverse orientation) was acquired to localize the lesion. For anatomical reference, a 3D T1-weighted scan was acquired at each time point (TR/TE = 9.9/4.6 ms, flip angle = 8°, acquired voxel size = 1.0 × 1.0 × 1.0 mm^3^, reconstructed voxel size = 0.88 × 0.88 × 1.0 mm^3^, FOV = 224 × 168 × 160 mm^3^, transverse orientation). For BOLD (Blood Oxygen Level Dependent) MRI during execution of language tasks (828 scans per task) and a breath hold paradigm (253 scans; see below), a 3D PRESTO-SENSE sequence (TE/TR = 33.2/22.5 ms, acquisition time per image = 609 ms, flip angle = 10°, FOV = 256 × 224 × 160 mm^3^, voxel size = 4.0 mm^3^ isotropic, sagittal orientation) was used^14^.

#### fMRI language tasks

We used a picture word matching (PWM) and a semantic decision (SD) task for the fMRI assessments^9^. PWM consisted of the simultaneous presentation of a picture^15^ and a word. Subjects had to decide whether these matched, and had to respond by pressing the left (correct) or right (incorrect) button with the left (non-paretic) hand. During SD, subjects had to decide if a visually presented noun referred to an animal. A control condition for both tasks consisted of an arrow pointing left or right. Pressing the corresponding button was required. This condition was added to mask out non-language related activation, such as motor activity, visual processing and cognitive processes needed for decision making other than the specific language skills. Stimulus presentation was self-paced, in order to induce a constant effort corrected for possible altered proficiency during recovery. During rest blocks a fixation cross was presented in the middle of the screen. Both tasks consisted of six study blocks, six control blocks and six rest blocks of 28 s (46 scans) that were interleaved.

#### Breath hold paradigm

During the breath hold paradigm, subjects were cued to hold their breath after normal inspiration by showing a bar that decreased in size according to the time that was left. Our previous study in patients with chronic aphasia showed that not all patients can resist 20 seconds of breath holding^9^. Since robust signal changes could be observed when alternating 14 seconds of breath holding with normal respiration, and blocks longer than 9 s have been shown to elicit clear BOLD signal responses^16^, 14-second blocks were used. Respiration was recorded with a pressure-sensitive belt around the abdomen.

The datasets generated during and/or analysed during the current study are available from the corresponding author on reasonable request.

### Data analysis

We used SPM8 (Wellcome Department of Imaging Neuroscience; http://www.fil.ion.ucl.ac.uk/spm/) for data analysis. Using rigid body transformations, all functional runs at all sessions were realigned to each other and to the FLAIR and anatomical scans. The patients’ lesions were manually outlined on the FLAIR images obtained at the first session around 2 weeks post-stroke. The segmentation was used to calculate lesion volume, and to mask out the lesion for subsequent normalization of all scans to the Montreal Neurological Institute (MNI) template^17^. Functional images were smoothed with an 8 mm kernel (FWHM).

#### Analysis of activation response to language tasks

A generalized linear model (GLM) with a boxcar function convolved with a hemodynamic response function was used. A high pass filter with a cut-off period of 128 s was applied. Rigid body realignment parameters (six) were added to the design matrix. Besides, scans that were affected by motion were effectively excluded by adding finite impulse response (FIR) functions to the design matrix. Volumes affected by motion were identified by first high-pass filtering the time-series (cutoff 0.01 Hz.), then calculating the mean volume across the time-series, and then for each individual volume estimating the mean sums of squares (MSS) of the deviation from the mean volume. If the MSS of a volume exceeded twice the mean MSS of all volumes, the volume was marked as affected, and a FIR was added to the design matrix.

For each subject at each time point an activation map was created with the estimate of the contrast between the language condition and the control condition (language activation), and a map with the corresponding statistical values.

#### Analysis of vascular response to breath hold paradigm

In order to assess vascular reactivity in response to the breath hold paradigm, analysis was conducted at two levels, similar to the approach in our previous study^9^. For the first part of the analysis, grey matter area was segmented in all subjects and lesions were masked out based on FLAIR imaging data. Subsequently, the average signal time course within the grey matter of the (healthy) right hemisphere from all subjects was calculated during the whole breath hold paradigm period. This signal was converted to percent signal change, and filtered using a high-pass filter with a cut-off period of 56 s (double duration of task and rest block) using spm_filter. Then we looked for changes over time of this response in the control group, and for differences between patients and controls. For each control subject, the signal response at each session was correlated with the average signal time course from all other healthy controls at session 1 with Matlab (the Mathworks inc.). Similarly, each patient’s signal time course was correlated with the average signal time course from all controls at session 1. As a result a distribution of correlation coefficients was available for each group at each session. Since correlation coefficients were not normally distributed, values were rank transformed. Subsequently a generalized linear mixed effects model was performed on the rank-transformed correlation coefficients using R (https://www.r-project.org) for each group at each session to test group, time, and group-time interaction effects (lme4 package “Fitting linear mixed-effects models using lme4”^18^).

For the assessment of whole-brain differences in breath hold responsiveness, the average signal time course in the grey matter of the right hemisphere of each individual was used as the regressor of interest in a GLM analysis for that particular subject (conform a regular first level fMRI analysis) to get an estimate of breath hold responsiveness in all voxels (effect size) and a statistical value of the fit (t value). Because the time course of each subject was used as regressor for that subject, differences in amplitude between subjects could not be detected, since the amplitude of the signal was effectively normalised. Therefore, the amplitude of the signal was determined from the amplitude of the sine wave with the dominant frequency (as identified from Fourier transformation of the signal time course). Before entering the voxel-wise GLM, the regressor was normalized by multiplication with the inverse of this amplitude. As a result, for each subject and each session two brain responsiveness maps were created: one with an estimate of the size of the breath hold response in each voxel, and another with the corresponding statistical value of the fit.

#### Identification of regions-of-interest

For each language task separately, activated brain areas in the control group were extracted and used as regions-of-interest (ROIs). A one-sample t-test was conducted on the signal contrast associated with the language task versus the control condition over all sessions, thresholded at p < 0.001 (uncorrected) with a cluster size of at least 10 voxels (k ≥ 10). Effectively, the resulting language activation map contained all brain areas robustly activated over all sessions in the control group. Since activation of the visual cortex was present despite addition of a corrective control condition, especially for PWM, activation within the occipital lobe was masked out. Since aphasic stroke patients predominantly activate areas similar to healthy subjects in the undamaged parts of the left hemisphere and recruit contralateral mirror sites in the undamaged right hemisphere across a large variety of language tasks^19^, ROIs in the left hemisphere were also flipped to the right hemisphere. If resulting clusters overlapped with originally activated right hemisphere areas in controls, these clusters were combined.

#### Prediction of outcome

Prediction analyses were conducted using SPSS 19.0. Language outcome was defined as Language Performance at the last session at one year after stroke.

First, separate simple Pearson correlations were calculated between language outcome and several baseline characteristics, i.e. lesion volume, language performance in the subacute phase, and age, as well as between language outcome and the fMRI-detected amount of activated voxels (p < 0.001, uncorrected) in each ROI during each language tasks.

Second, linear regression was conducted using a forward stepwise selection with the same baseline characteristics to extract the most relevant variables. Adding extra regressors to the model would invariably lead to an increased explained variance. Therefore, a goodness of fit corrected for complexity of the model (Akaike Information Criterion (AICc))^20^ was used to select the best model.

Third, fMRI-detected language activation that was correlated to language outcome in the first step was separately added to the model defined in the second step in another linear regression analysis. Predictive power was calculated and the AICc was used to determine if the model with added fMRI-detected language activation was superior to the one with only baseline variables.

#### Regions-of-interest analysis

ROI analyses were done with R using the lme4 package^18^. For each subject and each ROI, numbers of activated and responsive voxels (p < 0.001 uncorrected) during language tasks and breath hold paradigm, respectively, were calculated.

To elucidate the possible etiology of changes of language fMRI responses over time, we related language activation signals to language performance and to cerebrovascular reactivity. Changes in language activation responses over time that were not correlated with language performance or cerebrovascular reactivity were considered to reflect non/specific intersession variability. Below follows a description of the analyses for the factors language performance, cerebrovascular reactivity and intersession variability. Separate tests were conducted for each ROI and each task.

1. Factor language performance: Only patient data were used in this analysis. A correlation analysis was performed between language-related brain activation and language function. As language performance measures, we used online language accuracy, the composite language score (Language Performance), as well as specific measures for each language task. For PWM, the average of the Z-scores of the comprehension subtask of the AAT and BNT was used, while for SD the average of the comprehension and written language subtasks of the AAT was used. A Spearman correlation analysis was performed on the transformed data from all sessions.
2. Factor cerebrovascular reactivity. First, similar to above, a Spearman correlation between rank-transformed breath hold responsiveness and language activity in each ROI over all sessions was conducted. Second, to investigate if changes in vascular responsiveness in the patient group could have been responsible for changes in language task-related responses, two GLMs were built with language activation in each ROI as dependent variable. Since data were not normally distributed, a transformation into ranks was performed. Group, time and group-time interaction were used as fixed factors. With the first model we assessed changes in language-related activation over time within both groups. The second model was identical to the first, with the addition of breath hold responsiveness as covariate. Brain areas that fulfilled the following criteria were considered to harbor a change in activation response that was associated with altered cerebrovascular reactivity: i) exhibiting a significant time effect with the first model, that disappeared in the second model (when adding breath hold responsiveness as covariate); ii) the breath hold responsiveness covariate in the second model was significant, and thus explained a significant amount of variance. The last criterion was added to prevent extracting time effects with a borderline p-value that moved from just below 0.05 to just above, without the breath hold responsiveness covariate explaining a significant amount of variance.
3. Factor intersession variability. All areas showing changes that could not be related to alterations in language performance or to cerebrovascular reactivity, as described above, were considered to be associated with test-retest effects, non-biological intersession variability, or brain reorganization not or indirectly related to language recovery.

### Statistics

Baseline characteristics of patients and controls were compared using Student’s t-tests and Fisher’s exact test. Changes of language performance over time in patients were assessed with a linear mixed effect model. Distribution of correlation coefficients for breath hold responsiveness was assessed with a linear mixed effects model. To correct for multiple comparisons, we applied false discovery rate (FDR) correction to results from the ROI analyses. A p < 0.05 was considered significant. Means are shown with standard deviation, unless otherwise specified.

## Results

### Subjects

**Figure 1:**
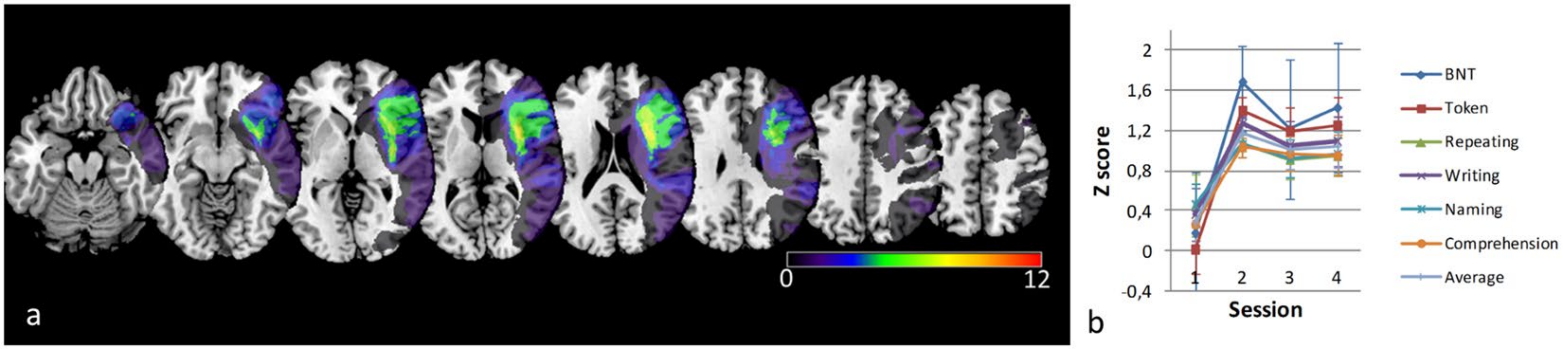
Lesion distribution and language performance scores of patients. (**a**) All lesions of patients are in the left hemisphere (radiologic orientation; left side is right hemisphere). Color indicates the number of overlapping lesions. (**b**) Language performance (Z-score, with a Dutch sample of aphasia patients as reference) on different subtasks of Aachen aphasia test and Boston naming task (BNT) over one year (within 2 weeks and at 3,6 and 12 months (sessions 1–4)).

Twelve patients (age 67.9 ± 11.4 years, ten males) and eight controls (age 65.9 ± 6.3 years, six males) were included. The location of the lesions is shown in Fig. 1A. Scores on the AAT and BNT at session 1, and type and severity of aphasia at the first and last sessions are shown in Table 1. The patients showed considerable recovery of language function over time (F(3,37) = 4.56, p = 0.008, Fig. 1B). Not all patients could be examined at all time points, due to various reasons, mainly tiredness or discomfort. One patient (#9) had a second stroke two months after inclusion and was not followed-up thereafter. For this reason, this patient only contributed to the baseline data. All twelve patients attended the first examination session. Time points of the last session for each patient are indicated in Table 1.

**Table 1:** Patients.

ID	Sex	Age (yrs)	Lesion location and volume (ml)	Language performance scores and aphasia type - first session								Language performance scores and aphasia type - last session									Time-point of last session
				Sp	BN	TT	Re	Wr	Na	Co	Type		Sp	BN	TT	Re	Wr	Na	Co	Type	
1	m	83	basal ganglia; 9	15		31	116	46	76	90	broca II		21	156	10	131	80	107	97	broca I	1 yr
2	m	68	lateral frontal; 30	19	108	36	140	71	59	98	broca II		30	167	0	148	90	119	112	anomic II	1 yr
3	m	63	lateral frontal; 89	17	125	28	141	67	73	76	broca II		23	164	8	145	88	111	109	unclass	1 yr
4	m	70	basal ganglia-insula; 15	19	116	30	130	63	80	97	broca II		26	160	18	141	84	109	113	anomic II	1 yr
5	m	74	temporo-occipito-parietal; 160	19	0	50	39	8	12	76	wernicke III		20	0	50	62	8	35	71	wernicke III	1 yr
6	m	69	middle frontal; 45	26	163	18	145	82	114	100	anomic		28	170	2	148	88	112	111	none	1 yr
7	m	62	fronto-temporo-parietal; 208	16	154	36	97	51	91	95	broca II		23	168	13	130	85	110	92	anomic	1 yr
8	m	66	lateral frontal; 68	24	96	48	141	45	81	49	wernicke II		26	125	23	131	81	104	84	anomic II	6 mos
9	f	77	lateral frontal; 92	29	108	23	147	90	94	71	anomic II										2 wks
10	f	53	lateral fronto- parietal; 70	18	0	43	5	3	0	61	global III		18	64	15	141	81	60	84	broca II	1 yr
11	m	86	temporo-partietal; 10	28	159	15	135	80	108	93	anomic		30	171	3	149	89	115	104	none	1 yr
12	m	46	basal ganglia- insula; 55	15	111	28	123	61	83	99	broca II		24	168	0	147	88	113	100	none	1 yr

ID Patient number, Sp Spontaneous language subtask of the Aachen Aphasia Test (AAT), BN Boston Naming Task, TT Token Test (higher scores reflect lower performance), Re Repeating subtask of AAT, Wr Written language subtask of AAT, Na Naming subtask of AAT, Co Comprehension subtask of AAT. Type Classification of aphasia with severity according to algorithm of AAT; I light, II moderate, III severe.

### Activated brain areas during language processing

**Figure 2:**
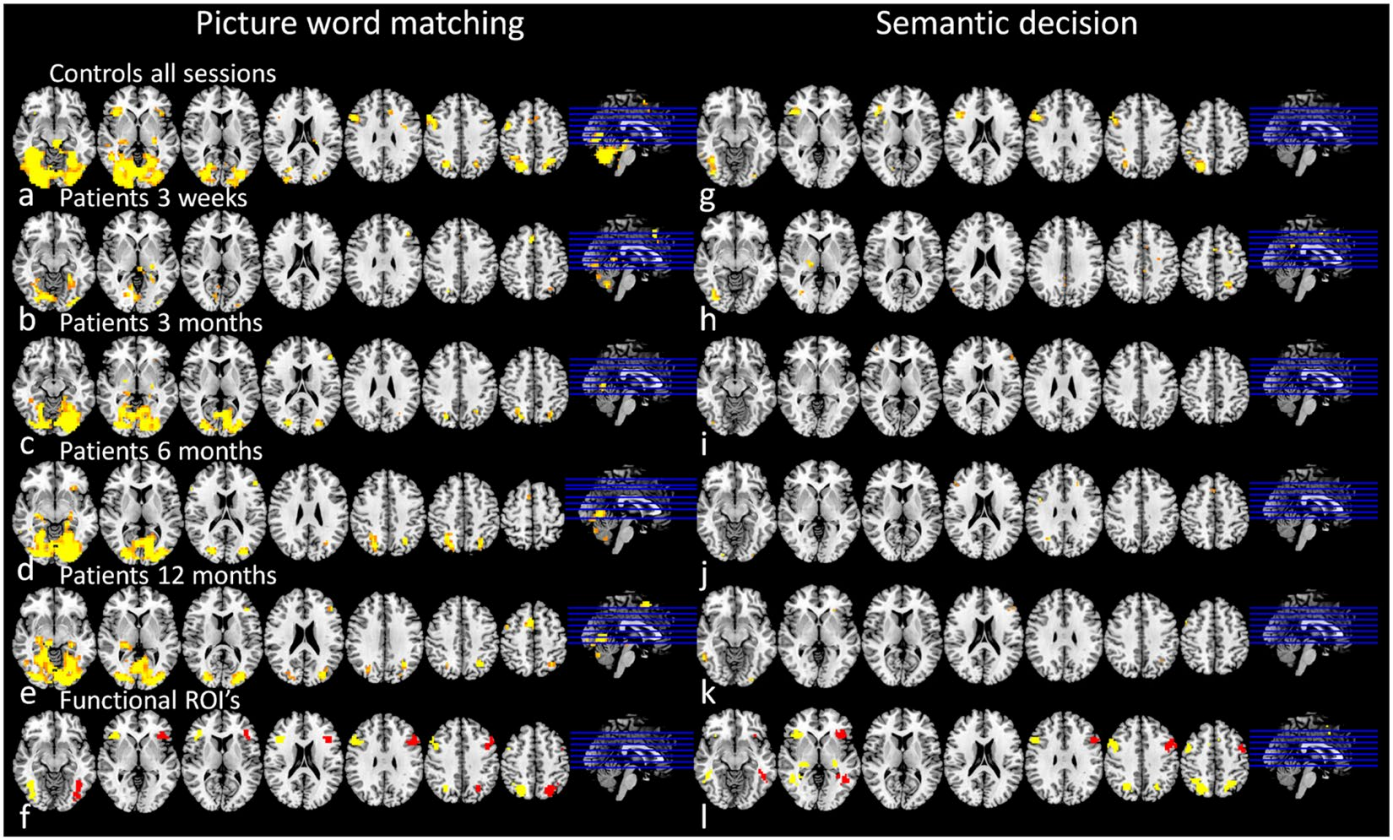
Brain activation during language tasks. Average brain activation pattern in response to language tasks (p < 0.001, uncorrected; radiological orientation; left side is right hemisphere). For picture-word matching: in controls averaged over all sessions (**a**), in patients at session 1 (**b**), 2 (**c**), 3 (**d**) and 4 (**e**); and ROIs for subsequent analyses (f: original clusters (yellow), flipped to right hemisphere (red)). For semantic decision: in controls averaged over all sessions (**g**), in patients at session 1 (**b**), 2 (**c**), 3 (**d**) and 4 (**e**); and ROIs for subsequent analyses (f: original clusters (yellow), and areas flipped to right hemisphere (red)). IFG Inferior Frontal Gyrus, R right, L left, Bil bilateral, MNI Montreal Neurological Institute coordinates (X, Y and Z), t t-statistic of peak voxel within cluster.

Average activation maps for language tasks in controls (over all sessions) and in patients (each session separately) are shown in Fig. 2. MNI coordinates^21^ and cluster sizes of activated areas in controls are shown in Table 2. The identified ROIs including right hemispheric homologous areas (mirrored from left hemispheric parts of the network) are shown in Fig. 2F and L. The PWM task was associated with activation of the inferior frontal gyrus pars triangularis/ opercularis, angular gyrus, and middle frontal gyrus in the left hemisphere; and the angular gyrus and inferior frontal gyrus pars triangularis in the right hemisphere. The lateral thalamus, dorsal anterior cingulate and a large part of the cerebellum and occipital lobe were activated bilaterally. During SD, the angular gyrus, inferior temporal gyrus and inferior frontal gyrus pars triangularis/ opercularis were activated bilaterally.

**Table 2:** Activated brain areas in healthy subjects.

Brain area	Side	MNI	Y	Z	t
		X			
Picture word matching					
Angular gyrus	R	22	−64	4	8.04
Lateral thalamus	R	26	−20	−6	7.61
IFG pars triangularis	R	34	32	−6	7.16
Dorsal anterior cingulate	Bil	6	12	46	7.01
Lateral thalamus	L	−22	−8	−6	5.75
Angular gyrus	L	−26	−64	42	14.84
Temporal occipital cerebellum	Bil	−34	−52	−14	22.81
IFG pars triangularis/opercularis	L	−38	24	−2	9.36
Middle frontal gyrus	L	−50	−4	46	10.41
Semantic decision					
Angular gyrus	L	−26	−64	46	12.48
Posterior inferior temporal gyrus	L	−34	−84	−10	13.47
IFG pars triangularis/opercularis	L	−50	16	30	10.82

### Prediction of language outcome

Language outcome of patients that were examined at one year (N = 10) correlated with language performance at session 1 (r = 0.57, p = 0.007) as well as with the amount of language-related activity in the left posterior inferior temporal gyrus during PWM (r = 0.65, p = 0.043) at session 1. As reflected by the correlation analyses, forward stepwise selection yielded a linear regression model with only language performance at session 1 as best model to predict language outcome (adjusted r^2^ = 0.57, F(1,8) = 12.9, p = 0.007, AICc = −11.118). Although predictive power increased somewhat when adding lesion volume and age, the goodness of fit (AICc) was lower (adjusted r^2^ = 0.70; F(3,6) = 8.08, p = 0.016, AICc = −7.4). Adding fMRI activity in the left posterior temporal gyrus during PWM to the model increased predictive power with an increase of the goodness of fit (adjusted r^2^ = 0.69; F(2,7) = 11.2, p = 0.007, AICc = −11.596).

### Relation between task-induced activation and language performance

Significant correlations between language activation and performance in the patient group were found for left and right ROIs during PWM. Language performance (composite score of off-line language tests) was associated with activation in the left posterior inferior temporal gyrus (r = 0.48, p = 0.0038) (Table 3). The average Z-score of comprehension and BNT was associated with right inferior frontal gyrus pars triangularis/operculars activation (r = −0.34, p = 0.043), and language accuracy during the online fMRI task was associated with activation in right posterior inferior temporal gyrus (r = 0.35, p = 0.036) (Table 3).

**Table 3:** Change of language activation over time, and presence or absence of relation with vascular reactivity or language performance.

Region of interest	Side	Control Change over time		Breath hold response		Stroke Change over time		Language performance		Breath hold response	
		Effect size	p	r	p	Effect size	p	r	p	r	p
Picture Word Matching task											
Dorsal anterior cingulate	B					0.0043	0.0002				
Angular gyrus	L					0.0023	0.0002				
IFG pars triangularis/opercularis	L	0.0015	0.0006			−0.001	0.0031				
Thalamus	L	0.005	0.0002								
Middel frontal gyrus	L	0.0012	0.0005			0.0018	0.0002				
Posterior inferior temporal gyrus	L	0.0009	0.0043			0.0041	0.0002	0.48*	0.0038		
Angular gyrus	R	−0.0011	0.0012			0.0029	0.0002				
IFG pars triangularis	R									0.58	0.0015
Thalamus	R			0.39	0.035	0.0018	0.0002				
Posterior inferior temporal gyrus	R	0.0014	0.0002					0.35**	0.036		
IFG pars triangularis/opercularis	R	0.0022	0.0002					−0.34***	0.043	0.38	0.045
Middel frontal gyrus	R	0.0029	0.0002			0.002	0.0002				
Semantic Decision task											
Angular gyrus	L	−0.0013	0.0004								
IFG pars triangularis/opercularis	L	0.0015	0.0002	0.40	0.030	−0.0002	0.0002				
Posterior inferior temporal gyrus	L					0.0018	0.0002				
Angular gyrus	R	−0.0028	0.0002			0.0014	0.0005				
IFG pars triangularis/opercularis	R	−0.0011	0.0015							0.39	0.038
Posterior inferior temporal gyrus	R	−0.0021	0.0002								

Summary of results of analysis of change of activation within the language system with or without relation to cerebrovascular reactivity and language performance (* composite score of all off-line language tests, ** accuracy of on-line task, *** combination of comprehension subtask of AAT and BNT). Change over time: Effect size (log odds) and statistical value (p, FDR-corrected) of linear fit through average activity in language areas (transformed into ranks) at each session over one year (<2 weeks, and 3, 6 and 12 months). Correlation with language performance (only in patients) with average activity within language area: r, correlation coefficient (Spearman) with p value (FDR-corrected). Correlation with breath hold response (both patients and controls) with average activity within language area: r, correlation coefficient (Spearman) with p value (FDR-corrected). IFG inferior frontal gyrus, B bilateral, L left, R right.

### Relation between task-induced activation and vascular reactivity

**Figure 3:**
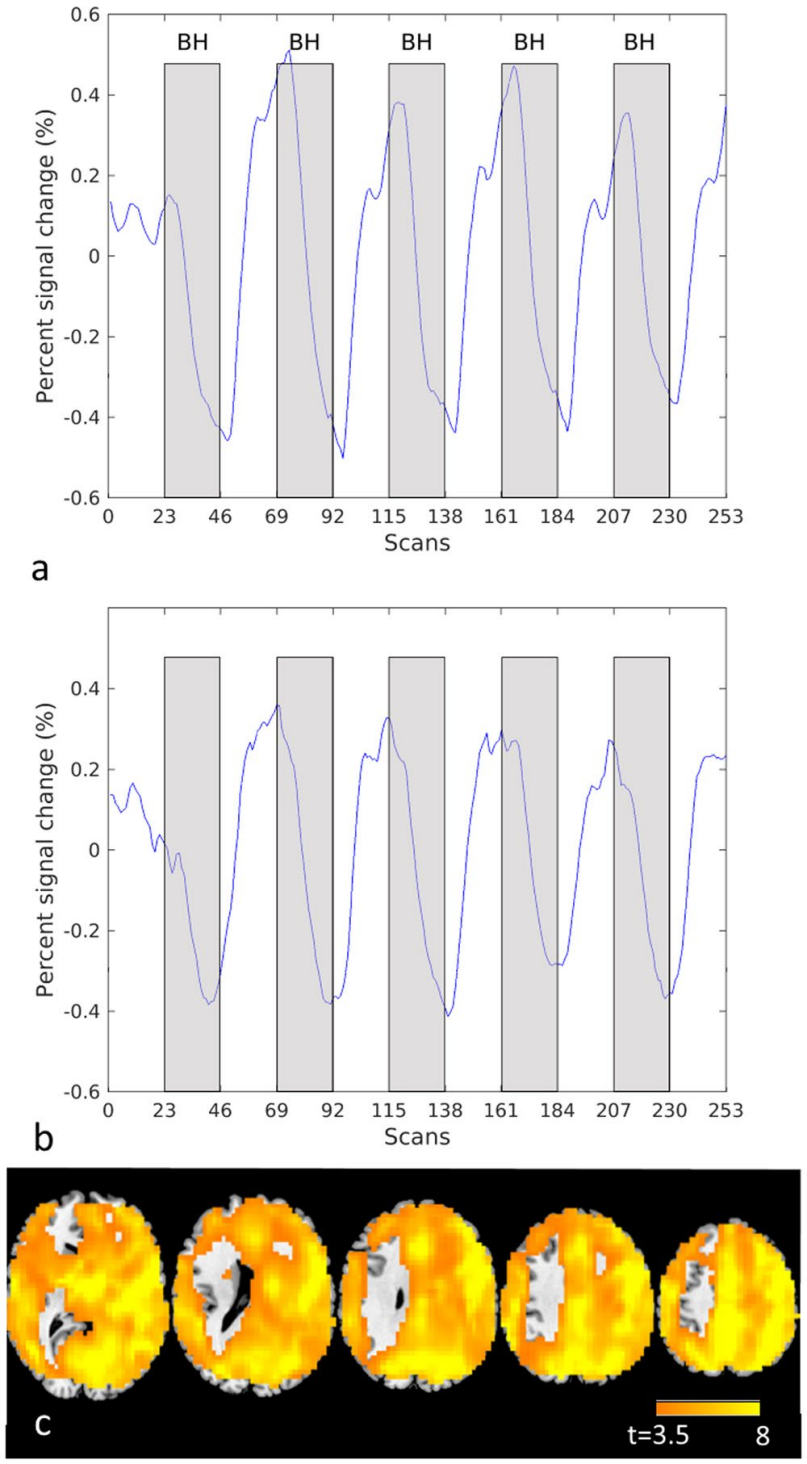
Breath hold responsiveness. Fitted BOLD signal change over time in the right hemisphere of healthy controls (**a**) and in patients (**b**). Statistical map showing vascular responsiveness (p < 0.0001, t > 3.5, uncorr) in controls based on correlation with the breath hold signal time course in the right hemisphere (left is left (c)).

The majority of patients successfully executed the breath hold tasks at sessions 1 (N = 9), 2 (N = 6), 3 (N = 10) and 4 (N = 10). The shape of the mean signal over time in grey matter of the healthy right hemisphere during execution of the breath hold paradigm (Fig. 3A and B) did not differ between patients and controls, nor was there a significant change over time (group effect: F = 0.75, p = 0.39; time effect: F = 0.024, p = 0.99; group-time effect: F = 1.0, p = 0.40). Amplitudes did not show differences either (group effect: F = 1.58, p = 0.21; time effect: F = 0.13, p = 0.94; group-time effect: F = 0.33, p = 0.81). Figure 3C shows the strong breath hold responsiveness of grey matter in the control group. As the average signal of the right hemisphere was used as regressor, stronger activation was measured in that part of the brain, as expected.

**Figure 4:**
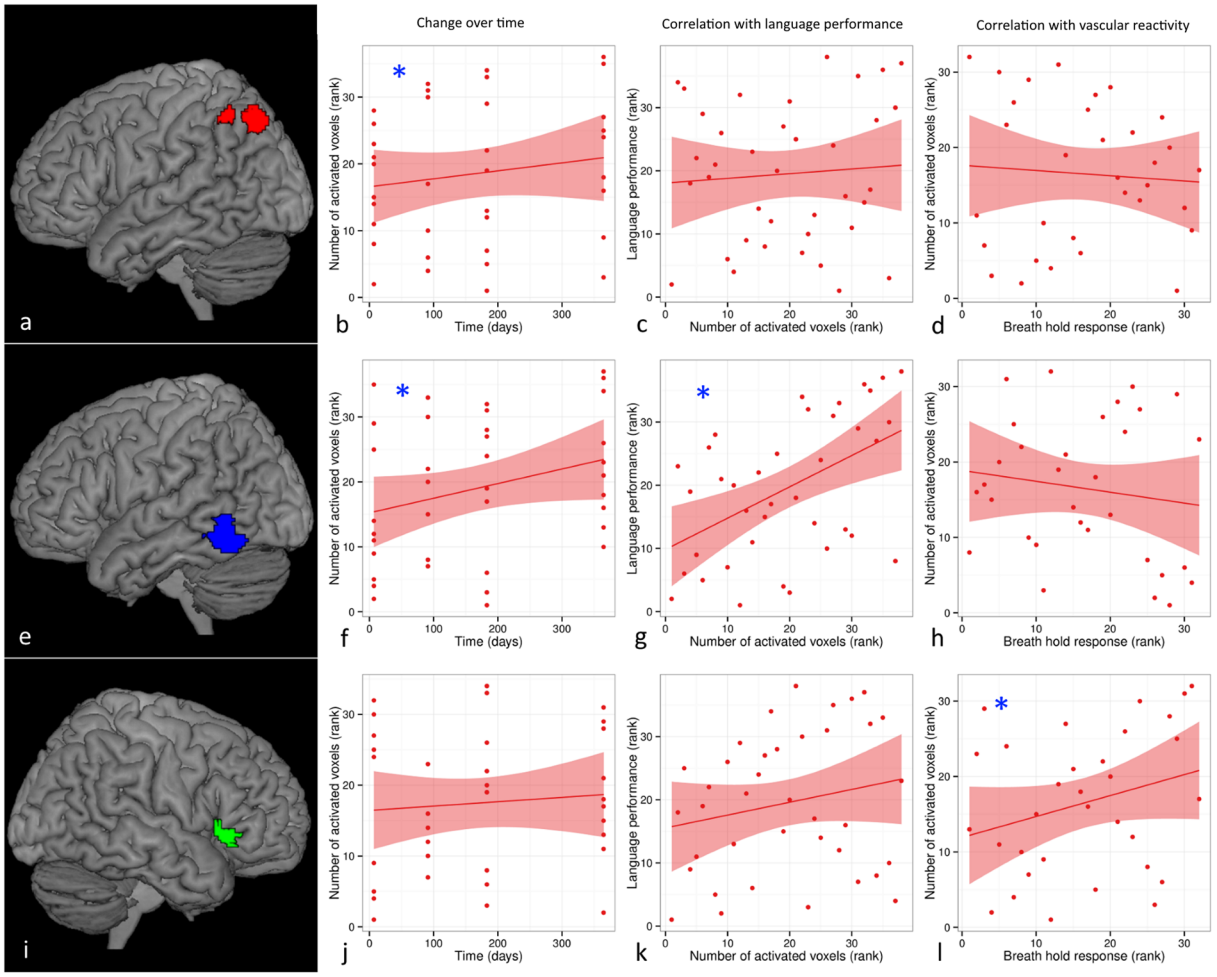
Example of change of language activation over time in relation to language performance and vascular reactivity in stroke patients. Significant change of language activation (induced by a picture-word matching task) over time was found in left angular gyrus (red overlay (**a**)) (**b**) and left posterior inferior temporal gyrus (blue overlay (**e**)) (**f**), but not in right inferior frontal gyrus pars triangularis (green overlay (**i**)) (**j**). Language performance was significantly correlated with language activation in left posterior inferior temporal gyrus (**g**), but not in left angular gyrus (**c**) or right inferior frontal gyrus pars triangularis (**k**). Language activation was significantly correlated with vascular responsiveness (measured with a breath hold paradigm) in right inferior frontal gyrus pars triangularis (**i**), but not in left angular gyrus (**d**) or left posterior inferior temporal gyrus (**h**). See also Table 3. Statistical significance is indicated by [*].

Significant positive correlations between breath hold responsiveness and language-related activation were found in patients in right inferior frontal gyrus pars triangularis/opercularis for PWM (r = 0.38, p = 0.045) (see also Fig. 4) and SD (r = 0.39, p = 0.038), and right pars triangularis for PWM (r = 0.58, p = 0.0015). In controls, positive correlations between hemodynamic responses to the breath hold paradigm and language tasks were present in right thalamus for PWM (r = 0.39, p = 0.035), and left inferior frontal gyrus pars triangularis/opercularis for SD (r = 0.40, p = 0.030).

Analysis of changes of language activation over time with correction for breath hold responsiveness revealed a breath hold-associated increase of language-related activation during PWM in right inferior frontal gyrus pars triangularis/opercularis of controls. No breath hold-associated change in language network activation was observed in stroke patients.

### Functional activation not related to changes in language performance or vascular reactivity

For both patients and controls, significant changes of PWM- and SD-induced activation over time were present in several ROIs, which did not show a significant correlation with either language performance or vascular reactivity (number of ROIs with significant change over time: 8 (controls, PWM), 5 (controls, SD), 8 (patients, PWM) and 3 (patients, SD) (Table 3) (see also Fig. 4).

Correlations with level of activity instead of number of activated voxels for the region-of-interest analyses show similar trends but were not statistically significant, presumably because of introduction of noise since ROIs in individual subjects partially overlap.

## Discussion

In this longitudinal study, activation of different parts of the language system was measured over one year and related to language performance and cerebrovascular reactivity in control subjects and aphasic stroke patients, which led to four main findings. First, prediction of language outcome is improved by adding the extent of activation of the left posterior inferior temporal gyrus during PWM in the subacute phase to a regression model including age, lesion volume, and language performance at baseline. Second, language (i.e. PWM)-related activation in the left and right posterior inferior temporal gyrus is related to improved language function, whereas activation in the right inferior frontal gyrus pars triangularis/ opercularis is inversely correlated to language recovery. Third, variations in language-induced BOLD activation in some parts of the language network can be related to differences in vascular reactivity (measured with a breath hold paradigm), as observed in the right inferior frontal gyrus in patients, and in the right thalamus and left inferior frontal gyrus in controls. Fourth, in both patients and healthy subjects several changes of language-related brain activation over time could not be directly linked to alterations in either language proficiency or vascular reactivity, reflecting unspecific intersession variability.

### Prediction of language outcome

Predicting outcome in individual patients with aphasia may support the development of new therapeutic strategies and the selection of patients in whom these therapies have the largest benefit. Several variables at the time of hospital admission are predictive of long-term outcome, such as age, lesion size and localization and initial aphasia severity^2,22,23^. Including information about the structural status of the language system, such as involvement of language areas in the lesion territory after stroke^24,25^, as well as structural integrity of right hemisphere white matter language tracts^26,27^. Our study shows that assessment of functional activation of intact language network areas can further help in outcome prediction. Saur *et al*. used a multivariate classification technique, with baseline characteristics such as age, language performance, and fMRI data from the subacute phase to categorize patients into “good” and “bad” recovery groups^4^. Activation of the right inferior frontal gyrus was most relevant in their model. In our study, activation of the left inferior temporal lobe in the subacute phase was associated with language recovery. This finding is in line with the concept that recruitment or preservation of key left perisylvian language areas is most important for recovery. However, we did not account for a non-linear relation between fMRI activation and outcome, which may partially explain the difference between the results of Saur *et al*. and ours. To further improve prediction, a classification algorithm such as that of Saur *et al*.^4^ could incorporate language function at baseline and data obtained with fMRI of frontal and temporal areas, DTI and structural MRI (lesion location and size).

### Plasticity of the language system

To assess changes in activation of different parts of the language network we selected a set of brain areas incorporating the majority of nodes of the network used for processing language, which were identified in control subjects and included homologous regions in the non-dominant hemisphere. Our approach was based on a meta-analysis of Turkeltaub *et al*. which demonstrated that the areas that are activated by a certain task in patients recovering from aphasia are similar to the ones activated in healthy subjects, with the addition of right hemispheric homologues in the inferior frontal and temporal lobes^19^. Therefore, we are confident that most if not all relevant nodes of the residual and recovering language network have been included in our analyses. Moreover, the activated areas that were identified are similar to the ones found in our previous study on recovery from aphasia in chronic patients^9,28^. Most consistently, recovery of aphasia has been attributed to upregulation of unlesioned left hemispheric language areas within the temporal and frontal lobe (see reviews^29–31^). This is supported by the association that we found between recovery of language function and increase in activation in the left posterior inferior temporal gyrus over the first year after stroke onset. The positive correlation of language function and activity in the posterior inferior temporal gyrus in both hemispheres may reflect upregulation of activity linked to reading^32^. However, this area may also contribute as part of a network for processing of higher order visual input. Alternatively, homologues brain areas in the right hemisphere and midline frontal structures such as dorsal anterior cingulate may contribute to language recovery by reflecting widespread upregulation of domain-general attentional and control processing^33–35^. The contribution of the right inferior frontal gyrus to language recovery after stroke is variable. Increase of activation of the right inferior frontal gyrus from the acute to the subacute phase of stroke is directly correlated with increase of language function^5^, and disruption of local neuronal processing in this region by TMS can worsen language performance in some, but not all patients^7^. On the other hand, TMS-induced inhibition of the right inferior frontal gyrus in the chronic phase has been shown to improve language performance^6^. The inverse correlation between language function and activation of the homologue of Broca in the right hemisphere, as found in our study, does not rule out the possibility of a contribution during separate phases such as the subacute one, as published previously, since it reflects an average effect over the whole first year of recovery. Besides, the ROI within the inferior frontal gyrus could actually consist of smaller separate adjacent areas that could not be separated with our approach. These parts may have a different role within the language network emerging at different phases during recovery. Indeed, inhibitory rTMS over the right pars triangularis has been shown to improve language production (increase naming accuracy and decrease latency), while inhibitory rTMS over the right pars opercularis can impede production^6,36^.

### Cerebrovascular reactivity

fMRI has been used extensively to probe plasticity of brain function after stroke. However, since stroke affects cerebrovascular reactivity, the coupling between blood flow and underlying neuronal activity, which is the basis of fMRI signals, may be disturbed in patients with cerebrovascular disorders. For example, task-related activation has been shown to be decreased in patients with cerebral amyloid angiopathy, which could be related in part to diminished cerebrovascular reactivity^37^. Indeed, in stroke patients with aphasia the hemodynamic response during execution of a language task can be altered^8^. In an earlier study we have shown that interpretation of the fMRI signal in the chronic phase after stroke is not confounded by altered vascular reactivity as measured through a breath hold paradigm^9^, although this may not hold for peri-lesional areas^38^. In the current study, we found that the vascular response in the right healthy hemisphere in patients was similar to that in controls at all time points. This supports the validity of using the temporal profile of this normal response in the right hemisphere for each subject individually to detect regional abnormalities (particularly the left hemisphere). The level of vascular reactivity correlated with the level of task-induced BOLD activation in the right inferior frontal gyrus in patients, and in the thalamus in controls. In healthy subjects change of language-related activation could be explained by a concordant change in vascular reactivity in several areas. Since no vascular events were noted in the healthy subjects, we assume that this reflects intersession variability rather than actual changes in vessel function. In stroke patients, however, no change of language related activity was explained by changes in vascular reactivity. This supports that also in earlier stages after stroke no significant misinterpretation is expected in fMRI studies in stroke patients. It should be noted that tasks were presented in blocks in our study. Signal time course analysis under these paradigm conditions is much less dependent on the hemodynamic response. When using event-related fMRI designs, however, interpretation may be biased to a larger extent. The assessment of vascular reactivity, preferably using breath holding while measuring end-tidal CO_2_, such as has been done previously^39^, is likely to overcome difficulties with interpreting breathold data in patients who may perform the breath hold task inadequately.

### Unexplained intersession variability

More than half of all language areas (13 of 18 in controls and 11 of 18 in patients), showed changes in task-induced activation over time that could not be related to either language recovery or alterations in vascular reactivity. The lack of a correlation with behavior may be due to a lack of statistical power or an indirect relationship with functional adaptations, but their occurrence in healthy controls suggests that such changes may not necessarily reflect post-stroke adaptive plasticity. Biological mechanisms such as a learning effect (test-retest variability) –which might be reflected by the reduction of activation in several right hemisphere areas during SD in control subjects– use of different cognitive strategies, unstable activity of the language network, and scanner-related variance, may be responsible. Yet, some caution is advised in the interpretation, since the results were not confirmed using whole-brain analyses. The substantial amount of intersession variability in healthy controls has implications for serial fMRI studies on (recovery of) language functions, in control as well as patient populations. This emphasizes the importance of incorporating a control group in longitudinal studies on functional recovery using fMRI in order to accurately distinguish specific disease-related changes in brain activation patterns from non-specific ones.

### Limitations

The relatively small sample size of the patient and control group and the fact that not all patients could attend all examinations may have masked effects by lack of statistical power and forced us to use a relatively liberal statistical threshold (but corrected for multiple comparisons). Activation of the occipital cortex during PWM was excluded in the analyses, as this most probably reflected visual processing that is inadequately masked out by the control condition. We cannot rule out presence of relevant activation in this part of the brain during this language task.

## Conclusions

Inclusion of functional activity of the language network in the subacute phase after stroke could aid in predicting language outcome in patients with aphasia. Changes of language-related activation over time in patients recovering from aphasia may reflect neuroplasticity within the language system or adaptations in cognitive strategy involving left and right hemispheric frontal and temporal language areas. A change in breath hold responsiveness over time was identified in healthy subjects, which suggests that part of the change in language-induced BOLD activation responses may be related to altered cerebrovascular reactivity. Moreover, changes in language-related activation not associated with either language recovery or alterations in cerebrovascular responsiveness were detected in several nodes of the language network in patients and controls. These may represent unspecific intersession variability. Our study supports the usefulness of controlling for external factors such as cerebrovascular reactivity and intersession variability in fMRI studies that aim to assess the etiology of activation patterns shifts in relation to function in patients with ischemic stroke.
